# KAER I/DD and GUIDE: Convergence to Increase Alignment of Beneficiaries with Intellectual Disabilities

**DOI:** 10.1093/geroni/igaf122.416

**Published:** 2025-12-31

**Authors:** Matthew Janicki, Kathryn Service, Jennifer Pettis, Lisa McGuire

**Affiliations:** University of Illinois Chicago, Rockport, Maine, United States; Rockport, Maine, United States; Gerontological Society of America, Washington, District of Columbia, United States; Gerontological Society of America, Atlanta, Georgia, United States

## Abstract

**Background:**

Adults with intellectual disabilities (ID) face significant barriers accessing dementia care, particularly in marginalized communities. To address these disparities, the National-Task-Group-on-Intellectual-Disabilities-and-Dementia-Practices (NTG) developed a grant-funded educational initiative linked to the Centers-for-Medicare-&-Medicaid’s Guiding-an-Improved-Dementia-Experience (GUIDE) Model to: (1) equip care navigators and practitioners with ID-specific knowledge and skills, and (2) increase rates of accommodation of Medicare beneficiaries with ID and dementia within GUIDE Participant programs. This initiative corresponded NTG technical expertise with the Gerontological-Society-of-America’s (GSA) Addressing-Brain-Health-in-Adults-with-Intellectual-and-Developmental-Disabilities: A Companion to the GSA KAER-Toolkit-for-Brain-Health (KAER ID/D), an adaptation of the KAER framework designed to improve dementia screening, diagnosis, and care planning for individuals with ID and other developmental disabilities.

**Method:**

As GUIDE participant sites launched in 2024 and 2025, the NTG created specialized training materials and technical assistance resources to enhance provider capabilities to enroll more beneficiaries with ID. Training modules covered three core areas: (1) information on ID (and related conditions) and dementia care planning, (2) caregiving histories and family dynamics in ID populations, and (3) connections with ID-focused service providers. Standardized, scalable education was delivered through learning management system training packs for GUIDE navigators and an online seminar series on dementia screening and diagnostics for practitioners.

**Outcomes:**

By integrating KAER ID/D principles, NTG ensured that GUIDE program personnel, particularly navigators, gained essential competencies in ID and dementia care. This training initiative enhanced the capacity of GUIDE agencies to provide equitable, informed, and effective support for adults with ID, ultimately increasing participation and improving care outcomes within this underserved population.

